# Circulating tumor biomarkers in early-stage breast cancer: characteristics, detection, and clinical developments

**DOI:** 10.3389/fonc.2023.1288077

**Published:** 2023-10-24

**Authors:** Jie Qiu, Da Qian, Yuancong Jiang, Liwei Meng, Liming Huang

**Affiliations:** ^1^ Department of Breast and Thyroid Surgery, Shaoxing People’s Hospital, Shaoxing, Zhejiang, China; ^2^ Department of Burn and Plastic Surgery-Hand Surgery, Changshu Hospital Affiliated to Soochow University, Changshu No.1 People’s Hospital, Changshu, Jiangsu, China

**Keywords:** tumor microenvironment, circulating tumor cell clusters, early breast cancer, biomarkers, detection

## Abstract

Breast cancer is the most common form of cancer in women, contributing to high rates of morbidity and mortality owing to the ability of these tumors to metastasize via the vascular system even in the early stages of progression. While ultrasonography and mammography have enabled the more reliable detection of early-stage breast cancer, these approaches entail high rates of false positive and false negative results Mammograms also expose patients to radiation, raising clinical concerns. As such, there is substantial interest in the development of more accurate and efficacious approaches to diagnosing breast cancer in its early stages when patients are more likely to benefit from curative treatment efforts. Blood-based biomarkers derived from the tumor microenvironment (TME) have frequently been studied as candidate targets that can enable tumor detection when used for patient screening. Through these efforts, many promising biomarkers including tumor antigens, circulating tumor cell clusters, microRNAs, extracellular vesicles, circulating tumor DNA, metabolites, and lipids have emerged as targets that may enable the detection of breast tumors at various stages of progression. This review provides a systematic overview of the TME characteristics of early breast cancer, together with details on current approaches to detecting blood-based biomarkers in affected patients. The limitations, challenges, and prospects associated with different experimental and clinical platforms employed in this context are also discussed at length.

## 1 Introduction

Cancer is a complex heterogeneous disease that is regulated by genetic, molecular, cellular, environmental, ethnicity-related, and socioeconomic factors. Currently, Breast cancer (BC) is the most frequently diagnosed tumor type in women and the leading cancer-associated cause of recurrence and mortality (1). Due to its global prevalence, many researchers have focused on gaining better exploration in cancer biology and developing tools for diagnosis and treatment. Over the past two decades, the average age of BC diagnosis has declined, and there have been concerted research efforts to define and explore the pathogenesis of BC including the roles of genetic susceptibility (2), DNA damage and repair (3), immunosuppression and immune evasion (4), and metabolic reprogramming driven by conditions such as hyperglycemia and obesity (5).

**Figure 1:**
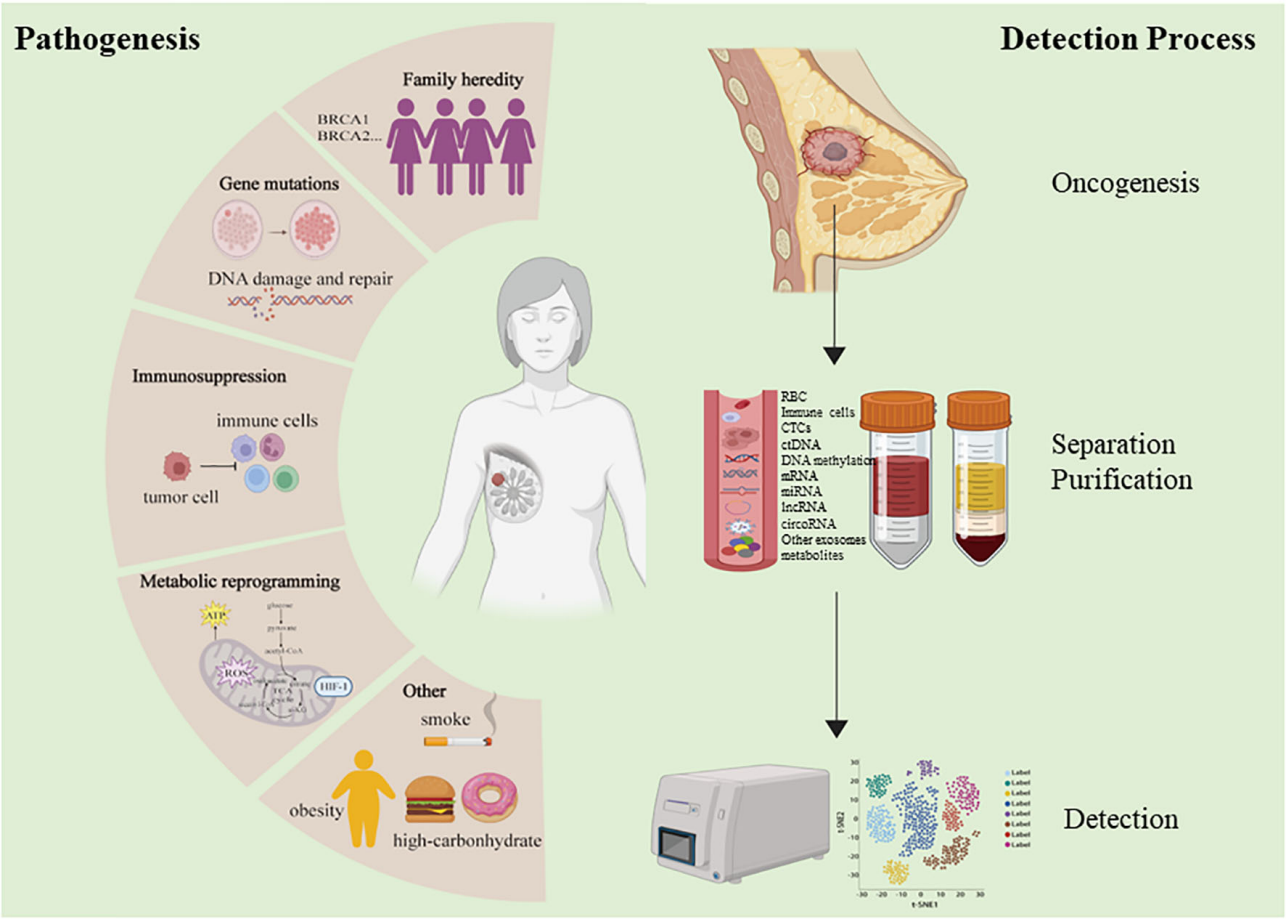
Mechanism of breast cancer and liquid detection process.

Mammography and ultrasonography are widely used to screen for early BC (EBC) (6). While noninvasive, these technologies are prone to high false negative rates and have limitations in terms of accuracy and sensitivity, and the diagnostic efficacy of common tumor markers in BC still has been relatively poor (7). Definitive BC diagnoses can only be confirmed through puncture or surgical biopsies, which are inherently invasive and risk overtreatment in cases where identified tumors are ultimately found to be benign. As such, there has been growing interest in the identification of more reliable and accurate noninvasive biomarkers that can aid in the detection of BC in its early stages and monitor tumor development through analyses of peripheral blood samples or other biofluids, as such biomarkers would offer clear clinical value (**Figure 1**).

In the past few years, a new diagnostic “liquid biopsy” has emerged and received widespread attention. The most common method is to collect samples of human peripheral blood for different analyses. Several other body fluids can also be used for specific biopsy applications, such as cerebrospinal fluid, pleural effusion, ascitic fluid and urine. Circulating tumor cells (CTCs) and circulating tumor DNA (ctDNA) are cornerstones of liquid biopsy (8). Besides, cell-free RNAs, antigen and metabolites, that encapsulated or not encapsulated in exosomes are also present in liquid biopsy specimens.

Our review provides a summary of circulating tumor biomarkers and cell properties associated with BC in blood, while also discussing the characteristic markers and metabolites that may aid in identifying affected patients. Recent advances in the detection and enrichment of circulating tumor cells (CTCs) are also discussed, and an overview of the potential clinical applications of these circulating tumor-related biomarkers is provided.

## 2 Breast cancer occurrence and development

In many cases, patient susceptibility to BC is thought to have a genetic component, with heterozygous high-penetrance variants in the *BRCA1* and *BRCA2* genes accounting for 50-80% of such variation and *BRCA1/2* gene sequences accounting for approximately 20% of cases of familial BC (9). Mutations in certain non-BRCA genes with low or intermediate levels of penetrance may also contribute to BC incidence and progression, including mutations in *TP53, PTEN, ATM, ESR1, CDH1, STK11, PALB2, RAD51*, and *BARD1 (* 2, 10, 11).

The DNA damage repair (DDR) system is responsible for detecting harm to the DNA induced by endogenous or exogenous factors, thereafter coordinating an appropriate signaling response to repair such damage. The DDR system also controls cell cycle progression to prevent the propagation of damaged DNA via cellular division, thereby protecting against oncogenesis or the differentiation of epithelial breast cells towards a more mesenchymal phenotype (12). BRCA1 helps to protect against DNA damage *in vivo*, thereby preventing the development of BC. Low levels of BRCA1 expression are evident in ~30% of BC patients owing to the methylation of the *BRCA1* promotor and/or the dysfunction of upstream signaling pathways responsible for its induction (13). BRCA2 can also help preserve genomic integrity through the promotion of homologous recombination-based DNA break repair, stabilizing stalled DNA replication forks, and regulating DNA damage-associated checkpoints in cell cycle progression (14). Other key proteins involved in DDR system-mediated cell cycle checkpoint regulation, DNA repair, and related processes include poly (ADP-ribose) polymerase 1 (PARP-1), the DNA-dependent protein kinase catalytic subunit (DNA-PKcs), ataxia-telangiectasia-mutated (ATM) kinase, and ATM and Rad3-related (ATR) kinase (15–17).

**Figure 2:**
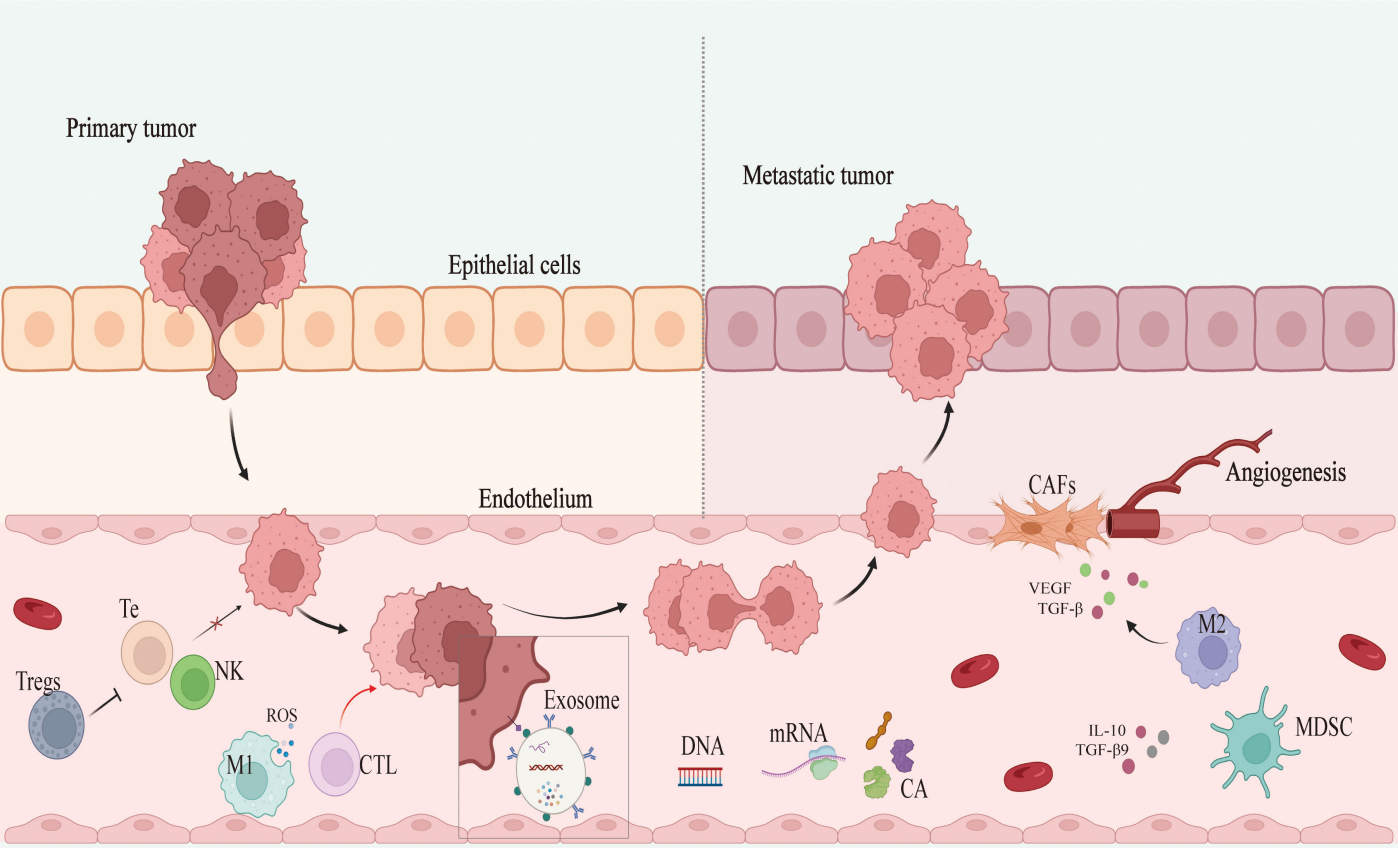
Mechanism of breast cancer development and metastasis. Tregs, regulatory T cells; Te, T-lympocyte; NK, nature killer cell; M1 and M2, M1 macrophages and M2 macrophages; CTL, cytotoxic T lymphocyte; CA, cancer antigen; MDSC, myeloid-derived suppressor cell; CAFs, cancer-associated fibroblasts; ROS, reactive oxygen species; VEGF, vascular endothelial growth factor; TGF-β, transforming growth factor-β; IL-10: interleukin10.

Solid tumor development can lead to the induction of a hypoxic, acidified, nutritionally depleted TME that can suppress immune cell activity and facilitate immune evasion, thereby hampering immune cell-mediated efforts to clear tumors and limiting the efficacy of immunotherapy (18–20). Tumor-associated macrophages (TAMs) are key members of the TME, with high TAM infiltration levels being tied to poor prognostic outcomes in BC (**Figure 2**) (21). Classically activated M1 macrophages are capable of producing reactive oxygen species (ROS) and recruiting cytotoxic T lymphocytes (CTLs), thereby facilitating adaptive immunity and tumor clearance (22). M2 macrophages, in contrast, can produce vascular endothelial growth factor (VEGF) and transforming growth factor beta (TGF-β) to promote tissue growth and angiogenesis, thereby supporting the growth of tumors (23). TME infiltration by regulatory T cells (Tregs) can protect tumor cells against immune-mediated clearance owing to their ability to secrete a range of immunosuppressive mediators such as TGF-β while also expressing inhibitory receptor molecules including CTLA4 that can interfere with NK and T cell functionality (24). Myeloid-derived suppressor cells (MDSCs) are a class of innate immune cells that also play key immunosuppressive roles in various tumors, generating ROS and utilizing amino acids otherwise necessary to support the proliferation of T cells. These MDSCs can produce IL-10 and TGF-β to suppress immune activity, and crosstalk between these cells, BC tumors, and other stromal cells can ultimately result in the enhancement of angiogenesis, invasivity, and metastasis (25, 26). Cancer-associated fibroblasts (CAFs) also comprise an important subset of cells in the TME and function by inhibiting the infiltration and activity of T cells, ultimately promoting other immunosuppressive cell recruitment and thereby modulating TME composition (19, 27).

Dysregulated metabolic activity is closely associated with the risk of BC development and can be observed in non-tumor cells ingredient within the TME. Obesity and diabetic individuals frequently exhibit elevated insulin and glucose levels together with the production of abnormal amounts of adipose tissue-derived adipokines, estrogens, and inflammatory factors (28). These alterations are particularly relevant in patients with postmenopausal hormone receptor-positive BC. Estrogen receptor (ER) signaling is related to the upregulation of genes important for fatty acid, glucose, and amino acid metabolism, whereas progesterone receptor (PR) facilitates the upregulation of genes related to the metabolism of cholesterol, steroids, lipids, fatty acids, nucleotides, and amino acids (29). In prior reports, glucose metabolism has been shown to promote the reprogramming of the hypoxic TME. Hypoxia contributes to an increase in the frequency of BC stem cells in a HIF-1-dependent fashion, and such HIF-1 activity is also related to chemoresistance (30). The highest levels of lipid metabolism-related gene expression for genes including FASN, CPT-A1, and PLIN1 have been observed in patients with HER2+ BC, whereas these levels are reportedly lowest in TNBC (31). The overexpression of ACLY, FASN, and SCD1 has been noted in individuals with HER2+ disease, whereas these genes are expressed at lower levels in TNBC (32). The amino acid requirements of tumor cells exceed those of healthy cells, with BC cells exhibiting altered glutamine, serine, glycine, and proline metabolic pathway activity levels, thus highlighting the potential importance of amino acid transport as a mediator of the proliferation of BC cells and the progression of this disease (33). Glutamine metabolism, in particular, is particularly as highly proliferative tumor cells utilize glutamine as a source of energy and a resource for the production of lipids, nucleotides, and proteins (34). These metabolic pathways ultimately complement one another, providing energy and modifying the TME in a manner conducive to BC progression.

## 3 Circulating tumor biomarkers

### 3.1 Circulating tumor cell clusters

CTC clusters consist of 2+ CTCs exhibiting stable cell-cell junctions, and these clusters are thought to exhibit a metastatic potential greater than that of a single CTC by a factor of 23- to 100-fold (35, 36). Most reports to date have determined that CTC clusters play an important role in the metastatic dissemination of advanced BC. These CTC clusters can evade immune-mediated detection, allowing for the blood-based transmission of tumors through the body (37). Whether these CTC clusters are present in EBC patients as they are in individuals with metastatic disease, however, remains to be studied in greater detail. Krol et al., however, successfully detected CTC clusters composed of at least 3 cells in EBC patients, indicating that these clusters may not be specific to advanced disease and suggesting a potential role for these clusters in the progression and spread of BC (38). Reduzzi et al. further employed the CellSearch approach to isolate CTC clusters exhibiting genetic abnormalities from 46 EBC patients, while using the ScreenCell method to assess CTCs from 23 EBC patients and 2 metastatic BC patients. Strikingly, clusters were found to be more commonly present in the blood of women with HER2-negative disease (39). Abnormal epithelial-mesenchymal transition (EMT) activity has also been linked to the metastatic progression of BC. Using microfluidic systems, researchers have successfully used antibodies specific for epithelial or tumor-associated antigens to capture CTCs, revealing high levels of EMT-related gene expression in these CTC clusters (40). As such, further research is warranted to explore the clinical significance of CTC clusters as predictors of distant metastatic progression and prognostic outcomes in BC patients.

### 3.2 Circulating tumor antigens

Circulating tumor antigens are closely associated with tumor cell proliferative, migratory, invasive, angiogenic, and immunomodulatory properties (35). Several such antigens have been detected in the serum of BC patients including carcinoembryonic antigen (CEA), CA15-3 (41), CA27-29, CA-125, Trop2 (42), tissue polypeptide specific antigen (TPS) (43), the circulating extracellular domain of HER2, and riboflavin carrier protein (RCP) (44). While these biomarkers can be dysregulated in EBC, their utility in the context of early disease screening faces challenges. The Videssa breast liquid biopsy approach (45) utilizes a combination of serum biomarkers and clinical factors to detect breast cancer with 93% sensitivity and 98% negative predictive value (NPV) in women 25-75 years of age, although in women under 50, it exhibits 87.5% sensitivity and 99.1% NPV (46). This technique thus required further development to reliably detect solid breast tumors. Future efforts to combine analyses of these or other circulating tumor antigens with results derived from ultrasonography, mammography, and imaging may also improve EBC detection rates.

### 3.3 Circulating tumor DNA

ctDNA has also been evaluated as a promising biomarker candidate that consists of small DNA fragments distinct from the nucleic acids that can be detected in live CTCs (47). Importantly, ctDNA levels and detection frequencies in plasma samples exceed those of CTCs in some reports (47, 48), although there have been some conflicting results (49).

In one report, an estimated 73% of BC patients were found to be positive for ctDNA prior to undergoing neoadjuvant chemotherapy (NAC), whereas these rates declined over the course of NAC treatment. Strikingly, ctDNA-positive patients were found to be significantly more likely to exhibit residual disease following NAC treatment, with all patients achieving pathological complete response (pCR) ultimately being found to be negative for ctDNA (50, 51). In the BRE12-158 trial (NCT 02101385), the detection of CTCs and ctDNA in patients with early-stage TNBC was found to be independently related to disease recurrence (52). As such, detecting ctDNA may represent an effective means of predicting NAC efficacy and the odds of pCR, while also enabling the assessment of disease recurrence and metastasis.

DNA methylation is a form of epigenetic modification that can influence gene expression and may be altered early in the course of oncogenesis such that certain methylation patterns may represent viable biomarkers for early cancer diagnosis (53). Abnormally methylated DNA has frequently been detected in the plasma and serum of cancer patients (54). Uehiro et al. were able to design a novel system for the assessment of the epigenetic characteristics of isolated ctDNA to facilitate the screening-based diagnosis of BC (55). Gao et al. further utilized a whole-genome bisulfite sequencing (WGBS) approach to assess methylated ctDNA, providing a means of distinguishing among cancer subtypes in a manner with great clinical potential (56).

Chromosomal instability is an important hallmark of cancer, and low-pass WGBS approaches can enable the assessment of chromosomal instability in ctDNA samples, facilitating the detection of BC recurrence with greater accuracy than that afforded by CEA or CA15-3 (57). The amplification of chromosome 1q21.3, for example, has been shown to be significantly associated with early relapse in patients with BC (58). Analyzing ctDNA can also permit the detection of *TP53* mutations, and may enable efforts to screen for BC patients harboring mutations in *BRCA1* (59).

On the whole, ctDNA analyses offer a greater dynamic range and are more representative of tumor specificity than are CTCs. However, the half-life of ctDNA is relatively short. Detecting mutations and epigenetic modifications in these ctDNA samples can support the early detection of BC. However, ctDNA copy numbers and content levels in peripheral circulation are generally low and the associated testing platforms are expensive, hampering the clinical application of ctDNA-based biomarker analyses at present.

### 3.4 Circulating noncoding RNAs

MicroRNAs (miRNAs) are short (19-23 nucleotide) transcripts that regulate a range of physiological and pathological processes via the modulation of gene expression (60). Circulating miRNA levels can be readily detected, and these levels are dysregulated in many pathological settings including cardiovascular disease (61), diabetes (62), obesity (63), and cancer. In one study, BC patients were found to exhibit increases in the levels of hsa-miR-21-5p and miR-106b-5p, whereas hsa-miR-205-5p and miR-143-3p expression was downregulated as compared to normal tissues (64). There is also evidence for the tumor type-specific roles of particular miRNAs. For example, miR-7 can promote B cell lymphoma development yet it inhibits BC progression, while miR-29 can promote BC but suppress lung cancer, and miR-16 can suppress hepatocellular carcinoma while driving the incidence and metastatic progression of glioma and lung cancer (65–68). MiR-21 has been shown to be among the most upregulated miRNAs in BC, and it can function by interacting with a range of target mRNAs including LZTFL1 and PTEN, thereby promoting the growth of BC cells (69, 70). There is also evidence for the ability of miR-155 to target BRCA1, thereby modulating DNA repair activity and progression through the cell cycle (71). MiR-205 can induce VEGF-A upregulation and ZEB family activity to facilitate BC cell growth and invasivity (72). While these results offer tantalizing glimpses of the pathologic relevance of these miRNAs, it is important to note that many miRNAs detectable in systemic circulation are not derived from tumors and instead originate from the steady-state activity of the other cells present throughout the body.

Long noncoding RNAs (lncRNAs) are > 200 nucleotides long and can serve as precursor forms of miRNAs or other RNA molecules, and can also interact with particular miRNAs through a competing endogenous RNA (ceRNA) mechanism. Certain lncRNAs have been shown to offer a high degree of utility as noninvasive biomarkers in particular cancers (73). Mechanistically, there is evidence for the ability of lncRNAs to control the proliferation, angiogenic activity, survival, invasivity, and metastatic progression of tumors through post-translational mechanisms and the remodeling of the chromatin (74). There have been several recent reports indicating that serum lncRNA levels can serve as biomarkers for BC diagnosis and prognostic assessment. For example, one group analyzed the serum levels of lncRNA-ATB, which can be activated by TGF-β, and FAM83H-AS1 in EBC patients and healthy controls, ultimately determining that lncRNA-ATB is superior to other tumor antigens such as CA15-3 when used to identify patients with stage I-II disease, whereas FAM83H-AS1 levels were related to tumor volume and metastatic progression to the lymph nodes (73). The dysregulation of the TINCR-miR-761 axis has been linked to the promotion of metastatic progression in early-stage triple-negative BC (TNBC) patients (75). Lu et al. further found that the lncRNA APOC1P1-3 was capable of decreasing levels of caspase-3/8/9 and PARP activity, overcoming Bcl-2 inhibition and specifically binding miR-188-3p to enhance the resistance of BC cells to anoikis, facilitating metastatic progression (76). Plasma lncRNA H19 levels are also reportedly significantly associated with ER status, PR status, C-erbB-2 levels, and lymph node metastasis in BC patients, with significantly higher presurgical levels in these patients as compared to analyses performed postoperatively (77). LncRNA H19 levels have also been found to be positively correlated with miR-675 expression, with miR-675 representing a potential lncRNA H19 derivative and a close documented association between lncRNA H19 and early HER2-positive BC (78).

CircRNAs are a subset of noncoding RNA exhibiting a closed covalent loop structure that is generated via the back splicing of pre-mRNAs (79). As they do not exhibit poly-A tails or 5’- or 3’- ends, these circRNAs are highly resistant to degradation mediated by RNase R or other exonucleases. Prior studies have explored the prognostic relevance of particular circRNAs in cancer patients. For example, one report observed the upregulation of circ-UBE2D2 in tamoxifen-resistant BC (80), while in another study the downregulation of circ-0025202 was evident in tamoxifen-resistant BC, with the upregulation of this circRNA suppressing the proliferative, migratory, and invasive activity of MCF-7 cells while enhancing their tamoxifen sensitivity and tendency to undergo apoptotic death (81). Zang et al. observed an increase in circRNF11 expression in paclitaxel-resistant BC with the silencing of this circRNA contributing to enhanced paclitaxel sensitivity in BC cells mediated via the miR-140-5p/E2F3 axis (82). Want et al. additionally determined that BC cells resistant to trastuzumab exhibit the upregulation of circ-BGN, which contributes to this chemoresistance through its ability to directly bind OTUB1 and SLC7A11, thus inhibiting ferroptotic cell death via the enhancement of OTUB1-mediated SLC7A11 deubiquitination (83). These studies suggest that efforts to monitor circRNA levels may provide particular value for the assessment and monitoring of BC patient responses to particular chemotherapeutic interventions.

Overall, these prior studies emphasize the potential utility of circulating miRNAs, lncRNAs, and circRNAs as effective noninvasive biomarkers that may facilitate the detection, prognostic evaluation, and monitoring of BC while also permitting analyses of the therapeutic efficacy of particular drugs (84).

### 3.5 Exosomes and metabolites

Initially discovered 40 years ago (85), exosomes were initially thought to represent a mechanism through which cells dispose of waste. However, more recent research has demonstrated that they are a subset of extracellular vesicles (EVs) that carry both membrane and non-membrane proteins and other macromolecular cargos including mRNAs, miRNAs, lncRNAs, DNA, lipids, and metabolites (86). Relative to normal cells, cancer cells reportedly release higher numbers of exosomes in response to the acidic and hypoxic conditions in the TME (87, 88). Paracrine signaling among BC cells in the TME mediated by exosomes can contribute to migratory, invasive, and metastatic activity and to immune evasion (89). Efforts to detect proteins present within BC-derived exosomes through analyses of blood samples can also support molecular subtyping efforts (90). Rontogianni et al., for example, were able to successfully differentiate between HER2+ BC and TNBC cases based on the proteomic analysis of circulating exosomes (90). Extracellular Hsp70 levels also reportedly offer value in predicting metastatic disease and therapeutic responsivity (91). Most studies of circulating exosomes to date, however, have largely centered on miRNAs. The exosome-mediated transfer of miRNAs between cells can enable them to directly regulate target mRNA expression within recipient cells (92). However, given the complexities and lack of standardization pertaining to the separation and collection of exosomes, analyzing them in the context of clinical trials remains challenging.

Metabolic reprogramming is a major hallmark of tumor development and growth, yielding a range of biological targets that are relevant to biomarker researchers and that may also have therapeutic implications (93). The TME of BC tumors is characterized by metabolic changes including glutamine addition, the Warburg effect, and elevated levels of lactic acid fermentation that help promote immunosuppression and angiogenic activity (94, 95). A subset of these cellular metabolites can be released into systemic circulation within EVs such that they can be detected in liquid biopsy samples. Accordingly, several reports have explored the potential application of these metabolites as biomarkers for early cancer patient diagnosis, characterization, and predictive treatment assessment (96). In their metabonomic analyses, Mao et al. determined that serum samples from patients with trastuzumab-resistant HER2+ BC exhibited higher levels of the essential amino acid L-arginine and the polyunsaturated membrane fatty acid arachidonic acid, both of which are important for immune system functionality (97). A prospective serum metabolomics study further determined that BC patients exhibited elevated levels of dimethyldodecane, galactose, and α-glyceryl stearate relative to healthy controls, while levels of glucopyranoside, tetradecane, mannose, and benzene 1,2 dicarboxylic acid were capable of differentiating among subgroups of BC patients based on staging, grading, and neoadjuvant status (98). In one study, the levels of four different metabolites in BC patient plasma samples (L-octanoylcarnitine, 5-oxoproline, hypoxanthine, and docosahexaenoic acid) were suggested to be associated with tumor pathological characteristics (99). Authors have also reported the utility of serum threonine, isoleucine, glutamine, and linolenic acid levels as predictive biomarkers of BC patient NAC responses (100), while Jobard et al. analyzed EBC and advanced BC patient blood samples and determined that circulating histamine, alanine, and betaine levels were elevated in EBC, suggesting that they may offer predictive value for patient staging (101).

While advances in targeted and non-targeted metabolomics analyses provide an opportunity to aid in the diagnosis and evaluation of BC patients, there are some limitations to the studies that have been conducted to date. Notably, patient-specific factors including hyperglycemia, drinking, smoking, and obesity can all complicate efforts to clarify the association between metabolite levels and BC status (102).

## 4 Emerging detection methods

Studies have suggested that CTCs offer value as potential biomarkers that can aid in the early diagnosis of particular cancers, while also enabling clinicians to monitor patients for minimal residual disease (MRD) (103). While CTC detection efforts thus hold immense diagnostic promise, the limited numbers of available CTCs pose a substantial technical challenge that is the focus of ongoing research efforts. Technologies including multiplex reverse-transcription quantitative PCR (RT-qPCR), imaging-based strategies, and microchip/microfilter devices can aid in the sensitive assessment of CTC samples present in biofluids such that disease status and therapeutic efficacy can be readily assessed (104). For example, the CTC Chip microfluidic device has been developed, employing antibodies such as anti-EpCAM (105), anti-CD146 (106), and anti-CD176 (107) for CTC capture. Automated cell imaging systems including the RareCyte and CellSearch systems, together with ScreenCell and CellSieve filters, can enable reanalyses of CTCs (39, 108, 109), improving detection rates for these rare tumor cells. In one study, authors successfully applied the CytoSorter system to enable CTC detection, and observed higher rates of CTC detection in cancer patients with advanced disease in a manner related to T staging, with detection rates as high as 100% in patients with T3/T4 disease (110). Krol et al. employed the CellSeed liquid handling platform and nanostructured titanium oxide-coated slides to achieve the shear-free detection of CTCs, successfully and efficiently capturing these cells from clinical samples while preserving their morphology to facilitate subsequent non-disruptive analyses (38). In addition, protein detection such as EpiSpot detection based on anti-cytokeratin (anti-CK) antibodies which is specifically combined with tumor specificity protein released by CTCs, is the most common detection approach (111).

The tumor cell-specific methylation status of ctDNA reportedly outperforms copy number variation levels as a predictor of BC patient risk. However, the reliable detection of such epigenetic modifications remains challenging owing to the low levels of ctDNA availability and the absence of on‐locus-specific DNA methylation technologies (112). To address these issues, researchers have leveraged digital PCR technologies to assess the ctDNA status of patients with various cancers, achieving successful ctDNA detection in 48% and >75% of EBC and advanced BC patients, respectively (113). In the c-TRAK TN trial (NCT03145961), digital PCR was utilized for the prospective monitoring of ctDNA in early TNBC patients or patients with residual disease following NAC as a means of guiding treatment efforts and demonstrating the clinical value of this detection modality (114). Gao et al. designed an improved ctDNA WGBS approach to allow for the unbiased assessment of ctDNA methylation status using small plasma sample volumes, successfully distinguishing among BC patients based on HR status (56). Yoshinami et al. were able to implement molecular barcode next-generation sequencing as a means of detecting ctDNA in EBC patients, thereby allowing for the identification of clinically relevant mutations with greater sensitivity than that afforded by traditional PCR (115). Epigenomic studies have also reported the use of Illumina arrays to prospectively evaluate samples with the goal of clarifying the association between blood DNA methylation and BC risk, as in the Sister Study (116) and EPIC-Italy study (117). In the I-SPY291 trial (NCT 27406347), researchers analyzed plasma samples from EBC patients prior to NAC and were able to use ultra-deep whole exon group sequencing to detect 16 patient-specific mutations in ctDNA, while also revealing that ctDNA clearance dynamics are related to NAC responses (50). These results may inform efforts to adjust patient treatment regimens in a timely and individualized manner.

As circulating miRNAs are small and present at low levels, their detection in a clinical setting remains challenging. Alternative detection strategies beyond RT-qPCR include microarray platforms capable of capturing large numbers of miRNAs, although these platforms tend to have a low dynamic range and are not capable of detecting novel miRNAs (118). Next-generation sequencing can facilitate miRNA detection, including novel miRNAs (119), but the resultant data require complex and time-consuming bioinformatics analyses to achieve reliable result interpretation. The digital molecular barcoding-based NanoString nCounter platform can allow for the precise quantification of exact miRNA copy numbers in a given sample, although only 800 miRNAs can be analyzed per slide (120). Hong et al. compared these different miRNA detection strategies and noted marked variations in speed, cost, and performance, revealing that miRNA-Seq can achieve a high degree of cost-efficiency when used for appropriate sample analyses (121).

Differential analyses of exosomal proteins associated with BC tumors and normal breast cells can offer insight into the processes that drive tumor progression (122). Risha et al. characterized the exosomal proteomes of MDA-MB-231 and MCF-10A cells when using three different exosomal separation techniques (ExoQuick, Ultracentrifugation, and Ultrafiltration-Ultracentrifugation) and detergents (n-dodecyl β-D-maltoside, Triton X-100, and Digitonin) through nano-liquid chromatography-tandem mass spectrometry (123). Liu et al. identified targets of interest through a droplet microfluidics digital ELISA strategy (124). There are also reports of the use of anti-CD81-modified immunomagnetic beads to separate EVs, thereby avoiding issues associated with precipitation and ultracentrifugation strategies (125, 126). Yu et al. employed a simple, sensitive, low-cost colorimetric aptamer sensor consisting of mucin 1 (MUC1) aptamers and a heme/G-quadruplex subunit with HRP-like activity that enabled the oxidation of substrates such that exosomes could be effectively detected (127). Nanoparticle Tracking Analysis (NTA) approaches can also provide insight into the characteristics of EVs based on light scatter following laser irradiation using the Stokes-Einstein equation and cell volume, permitting the detection of exosomes > 50 nm in size albeit with limitations pertaining to reliability and concentration measurements (128).

Metabolic dysregulation is a defining characteristic of most tumors (93), and a growing number of studies in recent years have focused on characterizing metabolic changes associated with different subgroups of BC patients. Mass spectrometry-based metabolic spectrum analyses offer a powerful means of detecting cancer-related metabolic changes that can offer insights into tumor pathogenesis and aid in the selection of candidate drug targets (129). In some reports, a combination of nuclear magnetic resonance (NMR) and gas chromatography-quadrupole mass spectrometry (GC-qMS) approaches have been used for metabolomic analyses of urine and breast tissue samples from BC patients and healthy controls, revealing that altered lactate, valine, aspartate, and glutamine metabolism are frequently observed in BC patients (130). Jasbi et al. were also able to successfully conduct metabolomic analyses through a targeted LC-MS/MS approach that revealed characteristic changes in arginine/proline metabolism, tryptophan metabolism, and fatty acid biosynthesis in EBC patients (102). Using nanoparticle-enhanced laser desorption/ionization mass spectrometry (NPELDI-MS), Huang et al. were able to rapidly detect serum metabolic fingerprints of BC, achieving high levels of accuracy (88%) and diagnostic efficiency in a manner that was reproducible and required very low amounts of serum input (131). These reports emphasize the rapid advancement of metabolomics-focused analytical technologies, and future efforts to integrate these platforms may improve the clinical utility of particular metabolites as biomarkers and therapeutic targets.

As summarized above, the detection of many circulating biomarkers faces many challenges pertaining to sensitivity, precision, and high costs. **Table 1** summarizes several common detection methods currentky used. Despite this, though, many clinical trials have explored the clinical relevance of these biomarkers when assessed in noninvasive liquid biopsy samples, and the US FDA has approved the detection of ctRNA and CTCs in BC patients (132). Future randomized clinical trials combining these noninvasive biopsy techniques with appropriate clinical interventions are expected to offer greater insight into the value of applying these detection strategies in routine practice.

**Table 1:** Dection methods and technical platforms for circulating markers.

Detection Method	Biomarker	Refence(PMID).
RT-qPCR/ digital PCR	CTCs, ctDNA, miRNA, lncRNA, proteins	35241469; 32170028
RareCyte system	CTCs	30277660
CellSearch system	CTCs, CECs	21737256; 31113842
CytoSorter system	CTCs	31908156
CellSeed system	CTCs	33762721
EpiSpot	CTCs, proteins	31330795; 24255082
WGBS	ctDNA, proteins	36474139
NGS	CTCs, ctDNA, miRNA	36613590; 32887501
Microarray	miRNA	34387660
NanoString nCounter	miRNA, EVs	27542126;
miRNA-seq	miRNA	37056771
MS	EVs, cellular metabolites, proteins	35302894; 30016454
ELISA	CTCs, Evs, miRNA, proteins	36081561; 29888919
NTA	EVs	31936142

RT-qPCR, reverse-transcription quantitative PCR; WGBS, whole-genome bisulfite sequencing; NGS, next generation sequencing; MS, mass spectrometry; ELISA, enzyme-linked immunosorbent assay; NTA, nanoparticle tracking analysis.

## 5 Clinical trials focused on circulating biomarkers in BC

Efforts to detect CTCs, ctDNA, miRNAs, and exosomes in peripheral blood samples have been used to assess the clinical treatment responses and prognosis of patients with breast cancer in several trials to date. Per the 2015 ASCO guidelines, ctDNA and CTCs are recommended as readouts for the monitoring of patient treatment responses (133). During the first-in-human study of oral SERD AZD9496 (NCT03236974), early changes in CTCs and ctDNA were explored as potential noninvasive tools, alongside joins up with paired tumor biopsies, to evaluate pharmacodynamics and early efficacy (134). In patients with locally recurrent unresectable or metastatic HR+/HER2- BC, next-generation sequencing-based analyses of tumor tissue samples or plasma ctDNA should be to detect mutations in *PIK3CA* in order to determine whether patients are eligible for treatment with the phosphatidylinositol 3-kinase inhibitor alpelisib plus fulvestrant (135). Clinical data supporting the routine use of ctDNA or CTCs for the monitoring of treatment responses in metastatic BC (MBC) patients, however, remain limited. Retrospective analyses of two phase III trials (NCT00253422; NCT00944918) revealed that in patients with *ESR1* mutations detectable in baseline analyses of ctDNA, fulvestrant treatment was associated with better progression-free survival (PFS) as compared to patients that had previously progressed on a nonsteroidal aromataseunhibitor (AI) (136). In the PALOM-3 trial (NCT01942135) (35), MBC patients with HR+/HER2- disease were randomly assigned to undergo treatment with the CDK4/5 inhibitor palbociclib plus fulvestrant group or placebo plus fulvestrant at a 2:1 ratio, with ctDNA samples being analyzed to detect mutations on day 1 and at the end of treatment. In this trial, data pertaining to *ESR1, PIK3CA*, and *TP53* mutation status were available for 331 and 195 patients on day 1 and at study end, respectively. Davis et al. were successfully able to apply whole exome sequencing to isolate and analyze ctDNA from 216 samples of plasma from 51 HR+/HER2- MBC patients in their phase II study of palbociclib plus letrozole or fulvestrant (NCT03007979). Their analyses revealed an association between higher blood tumor mutational burden and blood copy number burden (bTMB and bCNB) and both a lack of clinical benefit and poor PFS as compared to patients with lower bTMB or bCNB (all P < 0.05) (137). In the plasma MATCH trial (NCT03182634), MBC patients were separated into *ESR1* mutated, *HER2* mutated, *AKT1* mutated HR+, and *PTEN* mutated or *AKT1* mutated HR- subgroups, revealing that ctDNA analyses were able to rapidly and accurately facilitate such genotyping efforts, enabling the identification of rare mutations such that they represent viable therapeutic targets (138). In the BEECH trial (NCT01625286), dynamic ctDNA analyses were used to assess MBC patient PFS (139), although as this was a phase I/II study, additional evidence from a larger sample cohort will be necessary to validate this approach. Similarly, the APOLLO trail (NCT04501523) also utilized ctDNA to assess high-risk TNBC presents for recurrence, in order to provide effective follow-up treatment (140).

The above studies clearly indicated that the analysis of ctDNA samples as the clinically applicable biomarkers have been supported and can aid in efforts to predict patient treatment responses and prognostic outcomes, while also facilitating efforts to monitor mutational status in real time over the course of treatment, thereby providing a practical foundation for the application of personalized interventions. However, our review only limited describe the clinical application of detection ctDNA mutations, while other circulating markers researches are still very rare. Therefore, we look forward to the application of other markers in the diagnosis and monitoring of BC to help improve the efficiency of cancer treatment.

## 6 Final considerations

Liquid biopsy strategies and circulating tumor biomarkers have been major topics of interest in several studies published to date, and ongoing attempts are being made to assess the association between these biomarkers and BC development. Early-stage diagnosis and monitoring are particularly vital given that different subtypes of BC necessitate different treatment plans and exhibit different response rates, with important prognostic implications. No diagnostic technology has yet been established that is capable of reliably predicting the development of EBC or its clinical prognosis. Circulating biomarkers may aid clinicians in their efforts to understand the biological characteristics of patient disease, facilitating the design of more personalized treatment regimens and novel therapeutic methods based on the monitoring of tumor development and the identification of organs facing a high risk of metastases, potentially even enabling the detection of specific mutations that will allow for the adjustment of treatment in real time (141).

So far, the progress in liquid biopsy has been significant especially for non-small cell lung cancer (NSCLS), while plasma ctDNA is the most widely studied and widely used alternative to tissue tumor genotyping in solid tumors, and the detection of EGFR mutations in NSCLS is the first example to enter clinical practice (142). In BC, though liquid biopsy has the potential to help transform efforts to diagnosis and managament, they remain subject to many limitations at present. For one, current studies of CTCs and ctDNA have largely focused on their use as alternative indicators for use in the evaluation of patient hormone receptor or HER2 status. Most clinical trials conducted thus far remain in phase II or III, emphasizing a need for additional clinical validation. These biomarkers have also primarily been used to evaluate advanced BC patients rather than those with EBC, and research focused on the latter subgroup of patients will be necessary. Secondly, no circulating RNA targets such as miRNAs have yet to be implemented in clinical practice. However, the biological significance of cfRNA may be more extensive. An early prediction of cfRNA in preeclampsia has demonstrated the value of cfRNA in pregnancy abnormalities, endothelial cell disturbance and organ damage (143). Other study has identified tissue and subtype specific cell-free biomarkers in BC and lung cancer patients by characterizing cfRNA (144). But because of the low abundance of these miRNAs together with the costly and time-consuming methods needed to isolate them may ultimately serve as a barrier to their application. Even so, they remain invaluable research tools that can aid in studies of tumor cells and related pathological processes. EVs and metabolites are released into systemic circulation by virtually all cell types, harboring strong genetic signals and serving as facilitators of intercellular communication. The lack of standardized approaches to their isolation and detection, as well as uncertainties pertaining to the most effective reference genes, however, represent important challenges to their use. In addition, there have only been a few published reports in which clinical validation was performed, and additional studies will be important to gauge their clinical performance. In this article, we focus on the application of early detection of circulating markers in monitoring and evaluating the development of breast cancer. However, there is no evidence to suggest that blood biopsy can replace tissue biopsy in diagnosis and characterization. Therefore, if the weight of blood tests can be equivalent to tissue biopsy in the future, it will greatly reduce the treatment harm to patients. Due to the convenience and real-time of blood biopsy, it will be combined with imaging examinations to continuously update the development status of cancer, which is more conducive for clinical doctors to adjust and modify intervention measures in a timely manner.

In conclusion, ongoing research efforts focused on identifying further avenues for the application of these and other circulating biomarkers are expected to have profound implications for the detection, targeting, and immunotherapeutic treatment of EBC patients.

## Author contributions

JQ: Writing – original draft, Writing – review & editing. DQ: Validation, Writing – review & editing. YJ: Validation, Writing – review & editing. LM: Validation, Writing – review & editing. LH: Funding acquisition, Project administration, Resources, Validation, Writing – review & editing.
